# Research on factors affecting trust formation of generative AI agents in human–AI interaction contexts

**DOI:** 10.3389/fpsyg.2026.1886229

**Published:** 2026-07-16

**Authors:** Li Gong, Yaoming Gong

**Affiliations:** College of Publishing, University of Shanghai for Science and Technology, Shanghai, China

**Keywords:** generative AI, human-AI collaboration, perceived reliability, semi-technical users, structural equation modeling, trust formation

## Abstract

**Introduction:**

As generative artificial intelligence (GenAI) becomes deeply embedded in cognitive workflows, its inherent probabilistic generation mechanisms and the possibility of machine hallucinations may complicate the stability of semi-technical users’ trust. This study examined how semi-technical users’ interaction trust toward generative AI agents is associated with perceived explainability, perceived intention alignment, and perceived sense of agency, while also considering the moderating roles of perceived task complexity and domain self-efficacy.

**Methods:**

Survey data from 312 semi-technical users with experience in AI-assisted development were analyzed using structural equation modeling.

**Results:**

The results indicated that perceived explainability and perceived intention alignment were strongly and positively associated with perceived reliability, whereas perceived sense of agency showed only a small positive association with perceived reliability. Perceived reliability, in turn, was positively associated with human-agent trust. Notably, perceived reliability significantly mediated the associations between these two predictors and human-agent trust, whereas the indirect association involving perceived sense of agency approached but did not reach the conventional significance threshold and was therefore interpreted only as suggestive evidence. Furthermore, perceived task complexity positively conditioned the relationship between perceived explainability and perceived reliability. Domain self-efficacy also showed a significant positive moderation pattern in the relationship between perceived intention alignment and perceived reliability, which was opposite to the hypothesized negative direction.

**Discussion:**

These findings suggest that transparent and intention-aligned interaction designs may be especially important for supporting reliability-based trust formation in generative AI-assisted development, while user agency may require additional mechanisms beyond perceived reliability to account for its role in trust formation.

## 1 Introduction

In recent years, academic and industrial interest in artificial intelligence agents has grown rapidly (Feine et al., 2019), positioning them as increasingly important components of the modern technological ecosystem (Hughes et al., 2025). Specifically, large-language-model-based agents are reshaping human cognitive workflows, transitioning from theoretical exploration into large-scale industrial applications. According to the AI Index 2025 Annual Report (Maslej et al., 2025), the adoption rate of generative AI has doubled within a single year, with 78% of enterprises integrating it into their business workflows. Consequently, human-AI collaboration is no longer an experimental endeavor but has become an increasingly common productivity paradigm.

Despite their advanced cognitive capabilities, the probabilistic generation mechanisms of generative AI can make hallucinations difficult to eliminate. This phenomenon has been associated with declining public trust in AI innovation, a challenge that purely technical iterations may not adequately address. Furthermore, machine hallucinations may induce an illusion of competence among semi-technical users. In this study, semi-technical users are defined as technically adjacent, non-professional users with experience in generative AI-assisted development but without professional-level software development capabilities. Such users may over-trust the superficial fluency of AI outputs while neglecting critical review. This risk is particularly pronounced in AI-assisted development tasks characterized by high cognitive load and logical precision, where minor factual errors may be associated with software failures or workflow disruptions. Both over-trust and under-trust may be associated with the misuse or disuse of AI. Such patterns may create risks for critical decision-making (Ullrich et al., 2021). Although these users may rely on AI to compensate for technical skill gaps, their limited expertise may make it difficult for them to identify false code or logic flaws. Thus, establishing appropriate trust relationships between semi-technical users and generative agents has become an urgent challenge.

In this context, understanding how semi-technical users form trust toward generative AI agents becomes particularly important. Prior research on human-AI trust and appropriate reliance has suggested that users’ mental models, perceptions of transparency, and interpretations of system behavior play important roles in shaping trust judgments (Okamura and Yamada, 2020; Chiou and Lee, 2023; Naiseh et al., 2023). Related studies have examined mechanisms associated with users’ trust in AI systems, methods for supporting appropriate reliance (Zhang et al., 2020; Christoforakos et al., 2021; Li and Zhang, 2024), and approaches for evaluating trust-related states and behaviors (Choo and Nam, 2022; Yi et al., 2023; Jui et al., 2025). Moreover, scholars have noted that generative AI agents may require different interaction characteristics and feedback mechanisms across workflows. For example, medical research emphasizes empathy and explainable transparency (Deng et al., 2025), while the design field explores human-AI co-creation mechanisms (Fang et al., 2025). Ethical issues in the interaction process, including the amplification of algorithmic bias, credibility assessment mechanisms, and the risks of deception in social engineering (Zhang et al., 2025; Ou et al., 2026), have also received increasing attention.

In summary, although prior research has advanced the broader understanding of human-AI trust and appropriate reliance, the perceptual mechanisms through which semi-technical users form trust toward generative AI agents remain underexplored. Furthermore, existing research has often emphasized technical system features, while giving less attention to how users cognitively interpret observable interaction characteristics in relation to trust. To address these issues, this study adopts AI-assisted development—a complex human-AI collaborative scenario—as a point of entry. The present study focuses on the perceptual antecedents of trust. Specifically, it investigates how perceived explainability, perceived intention alignment, and perceived sense of agency are associated with perceived reliability and, in turn, human-agent trust.

## 2 Literature review

### 2.1 Generative AI agent interaction and trust formation

Since Joseph Weizenbaum created the first text-based computer program for human communication in 1966, AI agents have undergone a significant evolution from early text interfaces to contemporary voice dialogue systems and embodied conversational agents (McTear et al., 2016). These agents have transformed from primitive algorithmic models into highly complex and adaptive intelligent entities. Currently, academia is redefining the relationship between humans and generative artificial intelligence. For instance, Ordoumpozanis et al. (2025) argued that the non-deterministic output characteristics of GenAI render traditional user interfaces increasingly ineffective, necessitating the establishment of new interaction paradigms to accommodate the emergence of model capabilities. Regarding semi-technical users, research has found that when crafting prompts, they often lack accurate mental models of the system and erroneously apply human social dialogue strategies. This may be associated with speculative trial-and-error, making it difficult to bridge the gap between “user intent” and “effective model execution” (Zamfirescu-Pereira et al., 2023). Consequently, recent research has increasingly suggested that GenAI agents require innovative interaction methods to support more precise intention alignment and better realize their empowering potential.

As the human-AI relationship shifts from a tool-oriented to a partner-oriented dynamic, human-AI trust has become an important factor in the success of interactions (Ismatullaev and Kim, 2024). In the field of human-robot interaction, trust is defined as a user’s positive expectation and reliance on an agent’s ability to achieve specific goals within contexts characterized by uncertainty and vulnerability (Lee and See, 2004). Within the context of generative AI agents, investigating the latent factors associated with users’ trust judgments during the interaction process is particularly important (Shen et al., 2025). By definition, the user and the agent function as the trustor and the trustee, respectively (Deb et al., 2017). Trust is generally understood as being grounded in the user’s perception of whether the agent can successfully fulfill their objectives. In generative AI contexts, trust is also shaped by relational perceptions such as anthropomorphism, dependency, and privacy-related concerns (Mazhar et al., 2026a). This suggests that users’ trust in generative agents is not merely a response to technical performance. It also reflects how users interpret the agent’s relational role, potential risks, and reliance value during interaction. Therefore, this study adopts the following conceptual premise for trust formation: a user’s trust in an agent does not depend solely on the objective, underlying capabilities of the large model. Instead, it is grounded in the user’s perception of those capabilities derived from interface representations and interactive feedback. This process involves a subjective assessment in which users integrate their internal cognition with the system’s external manifestations throughout the interaction.

### 2.2 Model development

Following the theoretical discussion of agent intention interaction and trust formation, the underlying capabilities of large models are regarded as objective physical attributes. Because these attributes cannot be directly observed by semi-technical users, users’ evaluations of interaction trust may rely on cognitive interpretations of subjective perceived value. Previous research has indicated that AI representation alone does not directly enhance user trust; rather, this positive association may operate through mediating variables (Pelau et al., 2021). The current study regards users’ subjective perceived value as a critical factor related to trust formation. In development scenarios, agents may shape users’ cognitive evaluations through explicit interface features, where the interaction interface serves as an external representation of an agent’s intentions and reasoning logic. Consequently, this study defines human-agent trust in generative AI contexts as a dynamic state of reliance. This state is understood as being related to users’ integrated perception of both the interaction interface and the underlying model when they face uncertain probabilistic generation results.

#### 2.2.1 User perception characteristics

Because users cannot directly observe the underlying capabilities of generative AI systems, their trust judgments often rely on user-facing system attributes. In addition, such attributes have been associated with trust and subsequent behavioral intentions in generative AI use (Niu et al., 2026). Building on this view, the present study focuses on how semi-technical users interpret explicit interaction characteristics of generative AI agents. Specifically, perceived explainability, perceived intention alignment, and perceived sense of agency are treated as key perceptual antecedents associated with users’ reliability evaluations during AI-assisted development tasks.

Within the high-cognitive-load context of generative AI-assisted development, probabilistic generation mechanisms frequently induce significant perceived uncertainty and cognitive anxiety among semi-technical users. Current research in explainable artificial intelligence emphasizes that system transparency is the important basis for establishing healthy human-AI trust relationships (Zhang et al., 2023; Herrera, 2025). However, purely technical disclosure does not directly equate to cognitive transparency; system transparency needs to be transformed into user-friendly understandability to achieve substantive utility (Wang et al., 2024). If agents provide users with explanations characterized by logical depth and contrast, such perceived explainability may reduce information asymmetry between humans and machines. More importantly, it may be associated with users’ metacognitive accuracy and their construction of mental models regarding current AI capability boundaries during complex tasks (von Zahn et al., 2025). Through this cognitive engagement, users may develop greater confidence in the system’s ability to consistently output high-quality results, which may be related to stronger perceived reliability evaluations of the agent. Therefore, this study posits that a higher degree of perceived explainability is positively associated with perceived reliability among semi-technical users regarding the generated results.

Flathmann et al. (2025) noted that presenting agents as teammates rather than mere tools can enhance user acceptance. Furthermore, Schelble et al. (2025) demonstrated that trust collapse resulting from an agent’s intentional violation—failing to align with the user’s collaborative goals—is far more severe than that caused by a simple lack of capability. Semi-technical users’ evaluation of reliability depends on whether the system conforms to their intentions under specific constraints. When the system accurately aligns with collaborative goals through interactive feedback, this perceived intention alignment may serve as a goal-consistency cue associated with lower perceived risk of generative behavior (Mallick et al., 2024). Any autonomous AI behavior that violates human instructions may undermine the collaborative effectiveness of human-AI teams (Zhang et al., 2024). Thus, when an agent demonstrates a high degree of perceived intention alignment, semi-technical users are more likely to evaluate its outputs as reliable.

Within the human-AI collaboration paradigm, granting humans appropriate and effective control is a cornerstone for ensuring system trustworthiness (Shneiderman, 2020). This emphasis on control also resonates with recent discussions of generative AI as social actors, in which autonomy and empathy are treated as psychologically meaningful cues that shape users’ dependence on generative systems (Mazhar et al., 2026b). Granting users decision-making control in generative AI interactions may satisfy their psychological need for autonomy and support their understanding of system outputs. Such control has also been associated with positive reliability assessments (Westphal et al., 2023). Direct user intervention and iterative fine-tuning of an AI’s intermediate reasoning process may support users’ sense of agency and be related to more appropriate trust judgments during the identification and correction of machine hallucinations (Kim et al., 2026). When semi-technical users perceive a fine-grained control experience, they may be better able to monitor and adjust the agent’s outputs, which may be associated with stronger perceived reliability evaluations. Therefore, this study posits that a higher perceived sense of agency is positively associated with perceived reliability among semi-technical users.

Based on the theoretical logic described above, the following hypotheses are proposed:

*H1:* Perceived explainability is positively associated with perceived reliability.

*H2:* Perceived intention alignment is positively associated with perceived reliability.

*H3:* Perceived sense of agency is positively associated with perceived reliability.

#### 2.2.2 Trust building and the mediating role of perceived reliability

Existing research suggests that objective system characteristics are often interpreted through users’ perceived performance estimations before being associated with a trusting attitude (Lee and See, 2004). Other studies suggest that user trust in AI is built upon cognitive evaluations of its representational forms and capabilities (Glikson and Woolley, 2020). In development scenarios, perceived reliability represents users’ cognitive evaluation of whether an agent appears competent to execute complex tasks, which may then be associated with a trusting attitude toward the agent in uncertain contexts. Therefore, perceived reliability is treated in this study as an important cognitive link in trust formation. Based on this logic, explicit features such as perceived explainability, perceived intention alignment, and perceived sense of agency may be associated with human-agent trust through users’ rational assessment of system performance—namely, perceived reliability.

Based on the theoretical logic described above, the following hypotheses are proposed:

*H4:* Perceived reliability is positively associated with human-agent trust.

*H5:* Perceived reliability mediates the relationship between perceived explainability and human-agent trust.

*H6:* Perceived reliability mediates the relationship between perceived intention alignment and human-agent trust.

*H7:* Perceived reliability mediates the relationship between perceived sense of agency and human-agent trust.

#### 2.2.3 Task context and individual self-efficacy

Trust formation is not a static process but may vary dynamically according to task context. Task pressure may be associated with a shift toward heuristic cognition, resulting in a diminished ability to discern erroneous AI suggestions (Hermanns and Teubner, 2025). Consequently, when task complexity is high, the perceived cost of verifying AI results may be higher, and the relationship between perceived characteristics and reliability evaluations may become more salient. Users’ prior expertise may shape the dynamic game performance of human-AI collaboration (Ding et al., 2025). Domain self-efficacy represents users’ subjective confidence in their ability to evaluate generated content. Users with lower domain self-efficacy may rely more heavily on system intention alignment when forming trust. In contrast, users with higher domain self-efficacy may depend less on such perceptual cues because of their stronger independent evaluative capabilities (Inkpen et al., 2023).

Based on the theoretical logic described above, the following hypotheses are proposed:

*H8*: Perceived task complexity positively moderates the positive relationship between perceived explainability and perceived reliability.

*H9*: Domain self-efficacy negatively moderates the positive relationship between perceived intention alignment and perceived reliability.

**Figure 1:**
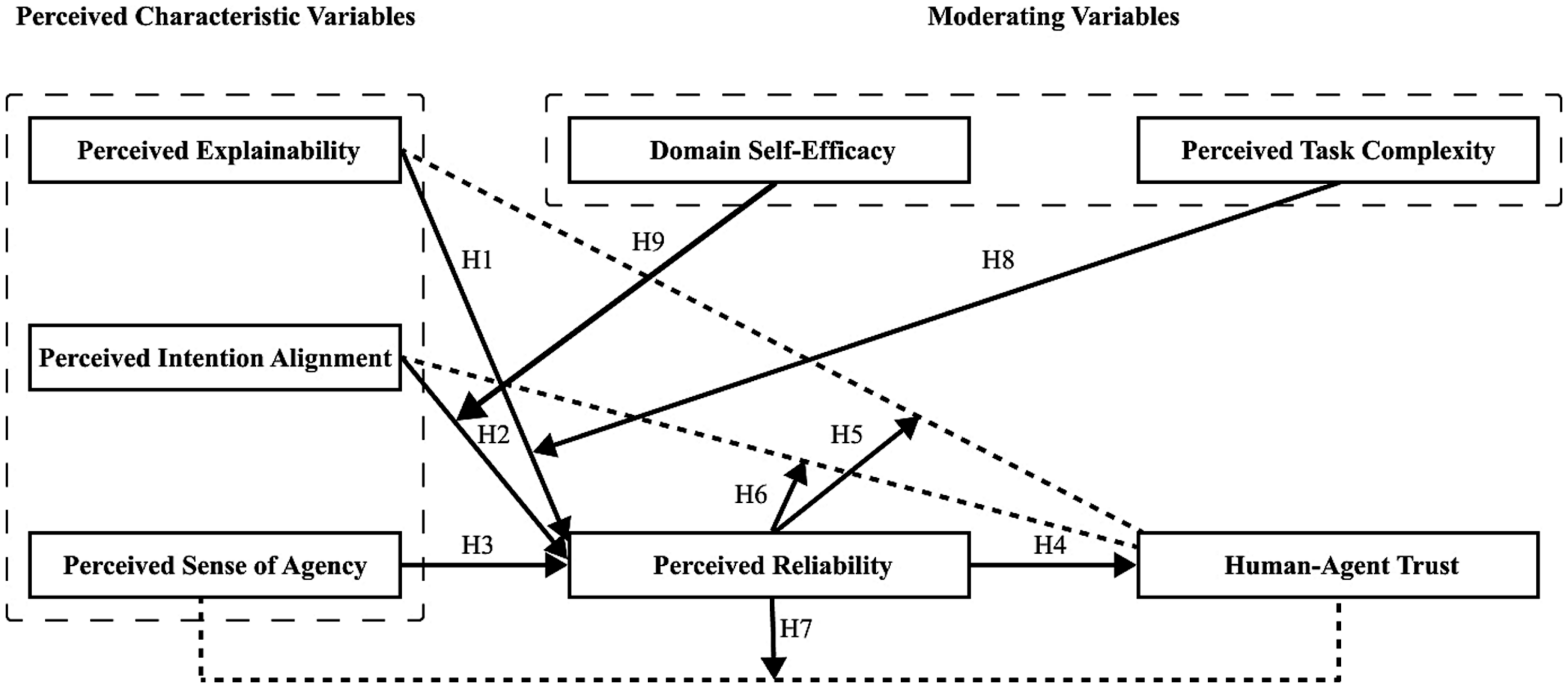
Research model.

Synthesizing previous research, the current study proposes a theoretical model as illustrated in Figure 1.

## 3 Methodology

### 3.1 Survey design

This study employed a questionnaire survey to collect data. All measurement variables were adapted from validated scales in established literature and contextually modified to align with the specific interaction scenarios of generative AI-assisted development. Each latent variable in the questionnaire consisted of three measurement items. A 7-point Likert scale was used for all items, with scores ranging from 1 (“strongly disagree”) to 7 (“strongly agree”). The specific variables and their corresponding measurement items are presented in Table 1.

**Table 1:** Description of questionnaire variables.

Construct	Code	Questions	Source
HAT	HAT1	Overall, I trust the outputs generated by the agent.	Jian et al. (2000)
	HAT2	I believe the agent is reliable when processing my tasks.	
	HAT3	I tend to rely on the agent’s recommendations when facing uncertain task outcomes.	
PR	PR1	I consider the code or text generated by the agent to have a high degree of accuracy.	Hermanns and Teubner (2025)
	PR2	The agent is capable of consistently producing logical results.	
	PR3	I believe there is a very low probability of the agent producing severe hallucinations or factual errors.	
PE	PE1	I can clearly understand the agent’s reasoning logic through interface cues or the output process.	Wang et al. (2024)
	PE2	When the agent provides a recommendation, it offers sufficient information for me to understand the underlying reasons.	
	PE3	The visibility of the agent’s workflow helps me understand its operational mechanisms.	
PIA	PIA1	The agent accurately comprehends and adheres to the specific constraints specified in my prompts.	Schelble et al. (2025)
	PIA2	The agent’s generation goals are highly consistent with my personal task intentions.	
	PIA3	In multi-turn dialogues, the agent demonstrates adherence to my collaborative goals rather than deviating from them.	
PSA	PSA1	I feel I have the freedom to intervene or interrupt at any time during the generation process.	Westphal et al. (2023)
	PSA2	I can conveniently make local modifications to specific parts of the agent’s generated results.	
	PSA3	Through the interactive interface, I can effectively control the direction of the final generated outcomes.	
PTC	PTC1	I typically use the agent for complex tasks that involve high cognitive load and strict logical requirements.	Hermanns and Teubner (2025)
	PTC2	The tasks I assign to the agent usually require significant mental effort to verify their correctness.	
	PTC3	The work I complete using the agent is subject to strict time constraints and trial-and-error pressure.	
DSE	DSE1	I possess sufficient domain expertise in the task areas for which I request assistance.	Inkpen et al. (2023)
	DSE2	I can independently and excellently complete tasks in this domain without AI assistance.	
	DSE3	I am highly confident in my ability to independently evaluate AI-generated results for domain-specific errors.	

* HAT = Human-Agent Trust, PR = Perceived Reliability, PE = Perceived Explainability, PIA = Perceived Intention Alignment, PSA = Perceived Sense of Agency, PTC=Perceived Task Complexity, DSE = Domain Self-Efficacy.

To identify participants appropriate for this research context, screening questions were included on the initial page of the questionnaire: “Have you used generative AI-assisted development tools in the past 6 months?” and “Do you possess professional software development capabilities?.” These questions were designed to identify participants who met the criteria of being semi-technical users with recent experience in AI-assisted workflows and without professional-level software development capabilities.

### 3.2 Data collection

The questionnaire was distributed through Wenjuanxing using a targeted recruitment approach. The survey was conducted in China and administered in Chinese. Participants were eligible if they had used generative AI-assisted development tools within the past 6 months and did not report professional-level software development capabilities. To encourage participation, a small monetary incentive was provided after completion of the questionnaire.

A total of 340 questionnaires were initially collected. Responses were screened for data quality before analysis. Invalid responses were removed if they failed the screening questions, showed duplicate submission records, had unrealistically short completion times, or displayed patterned responses such as invariant answers across scale items. After this screening process, 28 responses were excluded, resulting in 312 valid responses for analysis. Within the sample, males accounted for 54.2% and females for 45.8%. Among the valid responses, 78.1% reported non-intensive technical backgrounds, while the remaining respondents reported relatively stronger technical backgrounds. All valid respondents had recent experience with generative AI-assisted development and were not professional software developers.

### 3.3 Data analysis

This study proposed a hypothetical model to examine how explicit interaction characteristics of generative AI agents are associated with users’ micro-cognitive evaluations. The core antecedents and mediating variables include human-agent trust, perceived reliability, perceived explainability, perceived intention alignment, and perceived sense of agency, with perceived task complexity and domain self-efficacy serving as moderating variables. Given the complexity of the model and its focus on examining theoretically derived relationships, structural equation modeling was employed for data analysis. The data analysis process followed the two-step approach (Anderson and Gerbing, 1988).

First, the measurement model was tested using a progressive validation strategy. Because the measurement items were adapted from prior validated scales and modified for the generative AI-assisted development context, Cronbach’s *α*, KMO, Bartlett’s test of sphericity, and exploratory factor analysis (EFA) were used as preliminary diagnostics. Confirmatory factor analysis (CFA) was then conducted as the formal measurement validation procedure. Convergent validity was assessed using standardized factor loadings, composite reliability (CR), and average variance extracted (AVE), while discriminant validity was examined using the Fornell–Larcker criterion (Fornell and Larcker, 1981) and the heterotrait–monotrait ratio of correlations (HTMT). Common method bias was examined because all focal variables were collected through the same self-report questionnaire at a single point in time. Harman’s single-factor test was used as a preliminary diagnostic check. Given the limitations of this test, a one-factor CFA model including all observed indicators and an unmeasured latent common method factor model for the core SEM constructs were further estimated as CFA-based robustness checks (Richardson et al., 2009). The common latent method factor model was applied to the core SEM constructs because the moderation hypotheses were tested separately through regression-based interaction analyses using mean-centered composite scores.

Second, the structural model was evaluated and hypotheses were tested. After the measurement model showed acceptable reliability and validity, a structural equation model was constructed to test the path hypotheses. The overall goodness-of-fit of the model was assessed through a series of classic indices, including x^2^/df, RMSEA, CFI, TLI, and GFI. Following the attainment of acceptable fit indices, the direct main associations were examined based on standardized path coefficients and their levels of significance (*p*-values). Finally, mediation and moderation patterns were tested. For the mediating role of perceived reliability, this study utilized the Bootstrap non-parametric percentile resampling method, which offers higher statistical power (Hayes, 2017). For the moderating hypotheses, a complementary regression-based interaction approach was adopted via the SPSS PROCESS macro. Although core structural and mediation paths were evaluated using SEM, directly estimating latent interaction terms in covariance-based SEM significantly complicates model estimation and interpretation (Little et al., 2006). Therefore, to support clearer interpretation of interaction effects and simple slopes, mean-based composite scores from the CFA-validated items were used as manifest inputs. This approach was used to test the hypothesized moderation patterns while maintaining conceptual consistency with the established latent measurement model.

The structural equation modeling analysis was completed using IBM SPSS Amos 27.0, while the moderation analyses and simple slope tests were conducted using the SPSS PROCESS macro.

## 4 Results

### 4.1 Reliability, validity, and common method bias testing

As the measurement items were adapted to the context of generative AI-assisted development, a preliminary dimensionality screening was conducted before the formal CFA (Table 2). Cronbach’s *α* coefficients were first calculated to assess internal consistency. The results showed that the Cronbach’s α values for all latent variables ranged from 0.860 to 0.930, exceeding the acceptable threshold of 0.7. The overall KMO value was 0.834, and Bartlett’s test of sphericity was significant (*p* < 0.001), indicating adequate sampling suitability. EFA was used as a preliminary diagnostic check of the adapted items. The results showed that seven factors with eigenvalues greater than 1 were extracted, the cumulative variance explained reached 83.17%, and all items loaded primarily on their intended factors without severe cross-loading. These results suggested that the adapted items did not show obvious dimensional abnormalities in the current sample.

**Table 2:** Reliability and preliminary factor diagnostics.

Construct	Item	*M*	SD	Cronbach’s Alpha	KMO	Bartlett’s test (Sig.)	Eigenvalue	Factor loading
HAT	HAT1	4.163	1.012	0.860	0.834	0.000	2.345	0.787
	HAT2	4.237	1.015					0.781
	HAT3	4.186	1.032					0.780
PR	PR1	3.609	1.091	0.864		0.000	2.360	0.768
	PR2	3.644	1.066					0.771
	PR3	3.619	1.069					0.817
PE	PE1	4.747	1.115	0.902		0.000	2.510	0.877
	PE2	4.747	1.132					0.871
	PE3	4.788	1.051					0.860
PIA	PIA1	4.538	1.155	0.895		0.000	2.478	0.867
	PIA2	4.452	1.135					0.864
	PIA3	4.41	1.124					0.878
PSA	PSA1	4.276	1.224	0.918		0.000	2.578	0.913
	PSA2	4.234	1.221					0.937
	PSA3	4.266	1.238					0.914
PTC	PTC1	5.045	1.116	0.894		0.000	2.478	0.905
	PTC2	5.071	1.179					0.907
	PTC3	5.074	1.150					0.903
DSE	DSE1	3.33	1.338	0.930		0.000	2.633	0.935
	DSE2	3.388	1.358					0.931
	DSE3	3.33	1.309					0.931

To further evaluate the convergent and discriminant validity of the measurement model, confirmatory factor analysis (CFA) was conducted (Table 3). The composite reliability (CR) values for all latent variables ranged from 0.855 to 0.930, exceeding the recommended threshold of 0.7. The average variance extracted (AVE) values ranged between 0.663 and 0.817, surpassing the 0.5 cutoff point. Additionally, the standardized factor loadings of all measurement items were significantly greater than 0.6. These results suggest that the scale showed satisfactory convergent validity. Furthermore, the discriminant validity of the measurement model was assessed by comparing the square root of each latent variable’s AVE with the correlation coefficients between the variables (Table 4). The values on the diagonal represent the square root of the AVE for each latent variable, ranging from 0.814 to 0.904. Each of these values is strictly greater than the off-diagonal correlation coefficients in its respective row and column, supporting discriminant validity among the variables (Fornell and Larcker, 1981).

**Table 3:** Convergent validity.

Construct	Item	Standardized Loading	AVE	CR
HAT	HAT1	0.835	0.673	0.861
	HAT2	0.793		
	HAT3	0.833		
PR	PR1	0.815	0.663	0.855
	PR2	0.818		
	PR3	0.809		
PE	PE1	0.882	0.755	0.903
	PE2	0.881		
	PE3	0.844		
PIA	PIA1	0.852	0.739	0.895
	PIA2	0.875		
	PIA3	0.852		
PSA	PSA1	0.876	0.789	0.918
	PSA2	0.924		
	PSA3	0.864		
PTC	PTC1	0.850	0.740	0.895
	PTC2	0.869		
	PTC3	0.861		
DSE	DSE1	0.911	0.817	0.930
	DSE2	0.895		
	DSE3	0.905		

**Table 4:** AVE square root value.

Construct	HAT	PR	PE	PIA	PSA	PTC	DSE
HAT	0.821						
PR	0.662	0.814					
PE	0.492	0.633	0.869				
PIA	0.570	0.491	0.102	0.860			
PSA	0.232	0.006	−0.038	−0.052	0.888		
PTC	0.052	0.107	−0.026	0.019	0.058	0.860	
DSE	−0.140	−0.017	−0.040	0.019	−0.015	0.155	0.904

In addition to the Fornell–Larcker criterion, the heterotrait–monotrait ratio of correlations (HTMT) was calculated to provide a more stringent assessment of discriminant validity (Henseler et al., 2015) (Table 5). The HTMT values ranged from 0.016 to 0.663, all of which were below the conservative threshold of 0.85 recommended for assessing discriminant validity (Hair et al., 2019). In particular, although perceived reliability and human-agent trust are conceptually related constructs, their HTMT value was 0.663, remaining below the recommended cutoff. These results further suggest that the latent constructs in the model are empirically distinguishable, despite their conceptual proximity in the context of trust formation. This distinction is particularly relevant for HAT and PR, as HAT2 contains wording related to reliability. HAT primarily reflects a general expectation of agent dependability, whereas PR captures output-level reliability evaluations. Therefore, despite some semantic proximity at the item level, HAT2 was retained as a trust item rather than a perceived reliability item.

**Table 5:** HTMT ratios for discriminant validity.

Construct	HAT	PR	PE	PIA	PSA	PTC	DSE
HAT	—						
PR	0.663	—					
PE	0.497	0.633	—				
PIA	0.569	0.492	0.109	—			
PSA	0.232	0.040	0.041	0.058	—		
PTC	0.067	0.111	0.043	0.053	0.063	—	
DSE	0.140	0.039	0.045	0.052	0.016	0.158	—

Common method bias was further examined. As shown in Table 6, Harman’s single-factor test was first conducted as a preliminary diagnostic procedure. The results showed that the first unrotated factor accounted for 26.921% of the total variance, which was below the commonly used majority-variance criterion (Podsakoff et al., 2003). A one-factor CFA model including all 21 observed indicators was further estimated and compared with the theoretical multi-factor measurement model. The one-factor CFA model showed very poor fit to the data (x^2^/df = 17.048, GFI = 0.489, AGFI = 0.375, CFI = 0.311, TLI = 0.234, RMSEA = 0.227), indicating that the observed data could not be adequately explained by a single common factor. In addition, a common latent method factor model was estimated for the core latent constructs involved in the SEM-based structural and mediation analyses, including HAT, PR, PE, PIA, and PSA. The standardized loadings on the common latent method factor ranged from 0.49 to 0.61. Following the logic of latent method-factor modeling, the squared standardized method-factor loadings were used to estimate the proportion of variance attributable to the common method factor (Richardson et al., 2009). Accordingly, the estimated method variance ranged from approximately 24.01 to 37.21%. Because these estimates did not constitute a majority of the indicator variance, and because the one-factor CFA model showed poor fit, common method variance did not appear to dominate the measurement structure of the core SEM constructs. Nevertheless, common method bias cannot be fully excluded. Therefore, these results should be interpreted as robustness checks rather than as definitive evidence that common method bias was absent.

**Table 6:** Common method bias diagnostic results.

Diagnostic procedure	Scope	Key results	Interpretation
Harman’s single-factor test	All observed indicators	First unrotated factor explained 26.921% of total variance	Preliminary diagnostic; no dominant single-factor structure emerged.
One-factor CFA model	All 21 observed indicators	x^2^/df = 17.048, GFI = 0.489, AGFI = 0.375, CFI = 0.311, TLI = 0.234, RMSEA = 0.227	Poor fit; data not explained by a single common factor
Common latent method factor model	Core SEM constructs: HAT, PR, PE, PIA, PSA	The standardized loadings on the common latent method factor ranged from 0.49 to 0.61, corresponding to an estimated method variance of approximately 24.01–37.21%.	Some method-related variance may exist, but it did not appear to dominate the core SEM measurement structure.

### 4.2 Structural model testing and path analysis

The model fit was assessed to evaluate the alignment between the theoretical framework and the empirical data (Table 7). The goodness-of-fit indices indicated a satisfactory fit: x^2^/df = 1.529 (below the threshold of 3), and the Root Mean Square Error of Approximation (RMSEA) was 0.058 (below 0.08). Furthermore, both the Goodness-of-Fit Index (GFI = 0.951) and the Adjusted Goodness-of-Fit Index (AGFI = 0.933) exceeded the recommended benchmark of 0.9. Other indices also met the recommended criteria, suggesting that the proposed theoretical model showed an acceptable fit to the survey data.

**Table 7:** Model fit indices.

Fit index	x^2^/df	GFI	AGFI	TLI	PCFI	PNFI	RMSEA	CFI	NFI
Criteria	<3	>0.9	>0.9	>0.8	>0.5	>0.5	<0.08	>0.9	>0.9
Actual	1.529	0.951	0.933	0.947	0.799	0.770	0.058	0.959	0.963

**Figure 2:**
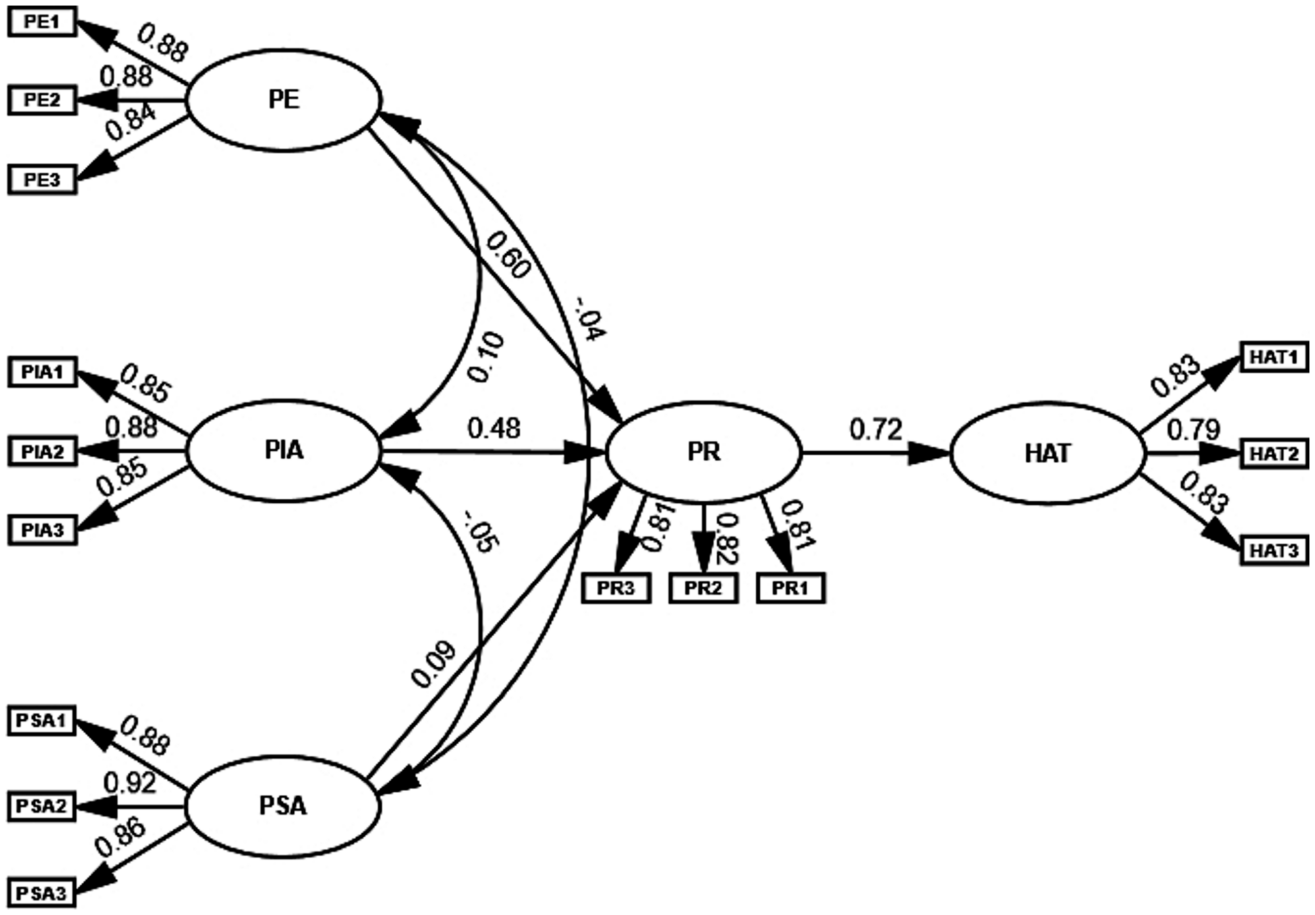
Final model with path coefficients.

The results of the path analysis are detailed in Table 8, and the final model with standardized path coefficients is illustrated in Figure 2. The path estimates showed that perceived explainability (*β* = 0.601, *p* < 0.001) and perceived intention alignment (*β* = 0.476, *p* < 0.001) were strongly and positively associated with perceived reliability, supporting H1 and H2. Perceived sense of agency also showed a small but statistically significant positive association with perceived reliability (*β* = 0.091, *p* < 0.05), providing limited support for H3. In addition, perceived reliability was significantly and positively associated with human-agent trust (*β* = 0.716, *p* < 0.001), supporting H4. Together, these results suggest that perceived reliability may serve as an important link between users’ interaction perceptions and human-agent trust.

**Table 8:** Results of structural equation model.

Hypothesis	Path	Standardized coefficient	CR	*p*-values	Result
H1	PE → PR	0.601	10.983	***	Supported
H2	PIA → PR	0.476	9.246	***	Supported
H3	PSA → PR	0.091	2.041	0.041	Partially Supported
H4	PR → HAT	0.716	11.251	***	Supported

***p < 0.001.

### 4.3 Mediation and moderation effects testing

To examine the mediating role of perceived reliability, this study employed the bootstrapping method with 5,000 resamples and a 95% confidence interval (CI). An indirect association is considered statistically significant if the CI does not include zero (Preacher and Hayes, 2008). The test results are presented in Table 9. For the pathways PE → PR → HAT and PIA → PR → HAT, the standardized indirect effect values were 0.431 and 0.341, respectively. In both cases, the 95% CIs did not contain zero and reached statistical significance (*p* < 0.001), suggesting statistically significant indirect associations through perceived reliability. Thus, H5 and H6 are supported. For the pathway PSA → PR → HAT, the standardized indirect effect was 0.066. The *p*-value was slightly above the conventional 0.05 threshold (*p* = 0.052), indicating that the indirect association did not reach statistical significance. Therefore, H7 was not supported. Nevertheless, given that the result approached the significance threshold, this pathway may be interpreted only as suggestive evidence of a weak indirect association rather than as a statistically confirmed mediation pathway.

**Table 9:** Results of mediating effect test.

Hypothesis	Mediation path	Standardized indirect effect	95% CI	*p*-value
H5	PE → PR → HAT	0.431	[0.3506,0.5076]	<0.001
H6	PIA → PR → HAT	0.341	[0.2604,0.4252]	<0.001
H7	PSA → PR → HAT	0.066	[−0.0003,0.1352]	0.052

**Figure 3:**
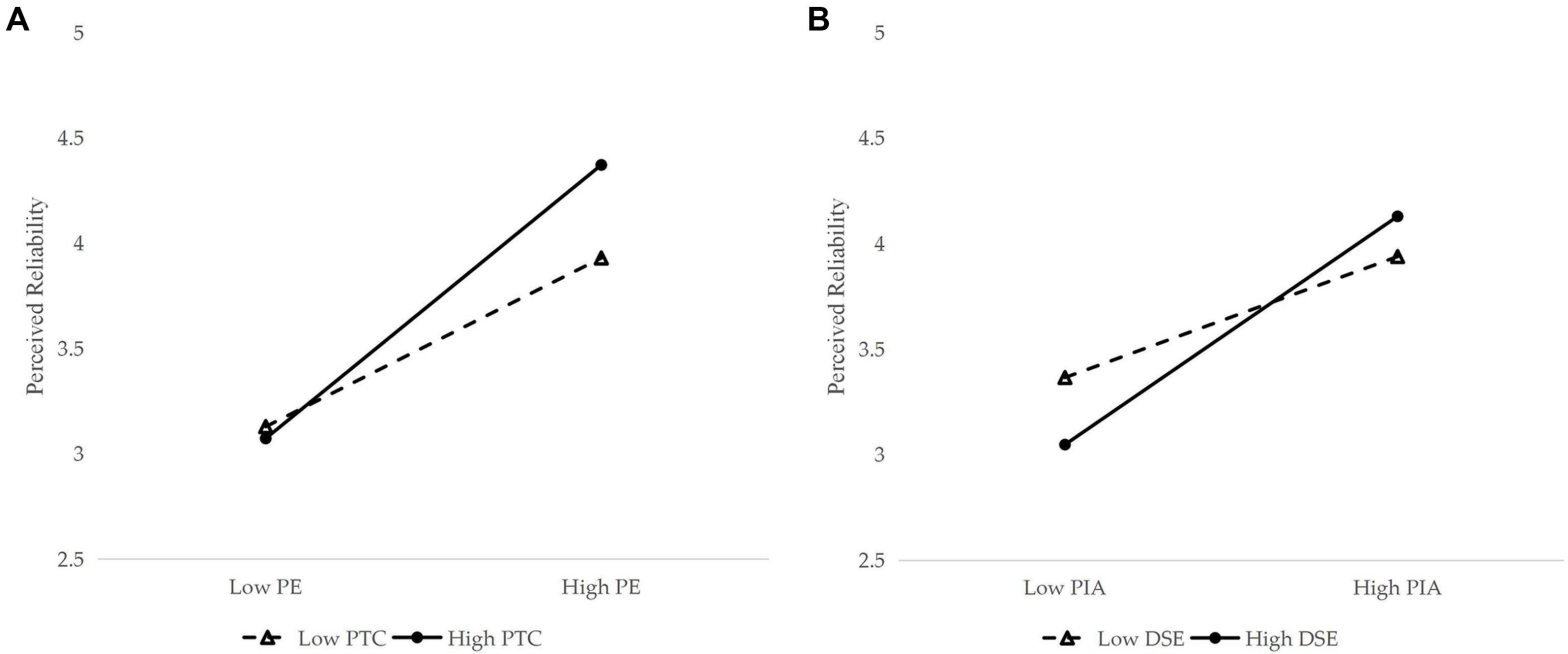
Moderating effects on perceived reliability: **(A)** Moderating effect of perceived task complexity; **(B)** Moderating effect of domain self-efficacy.

To test the hypothesized moderation patterns, regression-based interaction analyses were conducted using mean-centered composite scores for the relevant variables. The regression results are summarized in Table 10. The results showed that the interaction term between perceived explainability and perceived task complexity was significantly and positively associated with perceived reliability (*b* = 0.119, *t* = 2.913, *p* < 0.01). To further illustrate this interaction, a simple slope analysis was conducted and plotted (Figure 3A). Specifically, the simple slope results indicated that perceived explainability was positively associated with perceived reliability at both low and high levels of perceived task complexity; however, this positive relationship was stronger under high perceived task complexity (*b* = 0.646, *p* < 0.001) than under low perceived task complexity (*b* = 0.398, *p* < 0.001). These results suggest that perceived task complexity conditioned the relationship between perceived explainability and perceived reliability, supporting H8. In the second moderation model, the interaction term between perceived intention alignment and domain self-efficacy was also significantly and positively associated with perceived reliability (*b* = 0.098, *t* = 2.507, *p* < 0.05). As depicted in the simple slope plot (Figure 3B) and supported by the numerical results, perceived intention alignment was positively associated with perceived reliability at both levels of domain self-efficacy. This association was stronger for users with high domain self-efficacy (*b* = 0.522, *p* < 0.001) than for those with low domain self-efficacy (*b* = 0.277, *p* < 0.001). This significant interaction was opposite to the hypothesized negative moderation. Therefore, H9 was not supported. This suggests that domain self-efficacy may be associated with a stronger link between perceived intention alignment and perceived reliability, which warrants deeper theoretical discussion.

**Table 10:** Results of moderation regression analyses.

Model	Model 1			Model 2		
Predictor	PE	PTC	PE × PTC	PIA	DSE	PIA × DSE
Dependent variable	PR			PR		
*b*	0.522	0.092	0.119	0.400	−0.025	0.098
SE	0.044	0.042	0.041	0.047	0.039	0.039
*t*	11.86	2.181	2.913	8.529	−0.655	2.507
*p*	<0.001	0.030	0.004	<0.001	0.513	0.013
95% CI	[0.435, 0.608]	[0.009, 0.176]	[0.039, 0.199]	[0.307, 0.492]	[−0.102, 0.051]	[0.021, 0.175]
*R* ^2^	0.341			0.205		
Δ*R*^2^	—	—	0.018	—	—	0.016
Adjusted *R*^2^	—	—	0.335	—	—	0.197
*F*	53.197***			26.388***		
ΔF for interaction			8.483**			6.285*
VIF	1.007	1.002	1.007	1.000	1.004	1.004

* Coefficients are unstandardized regression coefficients based on mean-centered composite scores. ΔR^2^ and ΔF refer to the additional explanatory power contributed by the interaction term. VIF = variance inflation factor. ***p < 0.001, **p < 0.01, *p < 0.05.

## 5 Discussion

This section discusses how perceived explainability, perceived intention alignment, and perceived sense of agency are associated with users’ interaction trust in relation to perceived reliability. It also discusses how perceived task complexity and domain self-efficacy condition these relationships, with particular attention to the unexpected positive moderation pattern of domain self-efficacy.

### 5.1 Antecedents of trust formation and the mediating mechanism of perceived reliability

Specifically, perceived explainability showed a strong positive association with perceived reliability, whereas perceived sense of agency showed only a small but statistically significant positive association. This suggests that users’ ability to understand an agent’s reasoning logic and operational mechanisms may serve as an important basis for assessing its generative reliability. Theoretically, granting semi-technical users opportunities to participate in decision-making may support their reliability evaluation of the system. This result is consistent with Westphal et al. (2023), suggesting that system-provided explanations and decision-making control may reduce cognitive load and help users construct more accurate mental models of the system’s operational logic. This user group may face significant perceived uncertainty; if a system completely deprives them of control, they may reject its use upon discovering an error (Dietvorst et al., 2018). When semi-technical users perceive that the system provides reasoning steps in an understandable manner and allows for fine-grained local modifications, they may better understand and participate in the system’s logic. This involvement may reduce perceived loss of control arising from information asymmetry (Shin, 2021), thereby supporting their reliability evaluation of the agent during psychological assessment.

Furthermore, the significant positive association between perceived intention alignment and perceived reliability suggests semi-technical users’ underlying demand for the agent’s “goal consistency.” The reasoning behind this may be that as large models evolve from mere tools into highly autonomous collaborative agents, semi-technical users increasingly perceive them as digital teammates—social entities rather than simple instruments (Seeber et al., 2020). When a system functions as a teammate, users’ evaluation of reliability is no longer based solely on failure rates, but also on the degree of collaborative fit (O’Neill et al., 2022). In other words, users may be more sensitive to intention violations than to technical performance failures alone (Li and Lee, 2026). When an agent demonstrates high compliance and goal consistency across multi-turn dialogues, semi-technical users may view it as more capable of understanding and following their collaborative objectives. This alignment at the intentional level may be associated with greater confidence in AI task performance (Askell et al., 2021), which in turn may be related to stronger perceived reliability. Consequently, higher perceived intention alignment was associated with stronger perceived reliability.

Finally, the mediation analysis supported the PE → PR → HAT and PIA → PR → HAT pathways, whereas the PSA → PR → HAT pathway approached but did not reach the conventional significance threshold. Therefore, the mediating role of perceived reliability should be interpreted mainly in relation to perceived explainability and perceived intention alignment, while the agency-related indirect association should be treated only as suggestive evidence. For the supported pathways, this suggests that explicit system characteristics may not be directly associated with trust attitudes across cognitive stages. A plausible explanation is that semi-technical users, lacking the professional expertise to evaluate the underlying logic of AI, may rely on perceived reliability as an evidence-verification cue. The logical cues provided by perceived explainability and the goal-related cues associated with perceived intention alignment may serve as cognitive bases for perceived reliability. As noted by Lee and See (2004), in uncertain contexts, users may first establish a performance assessment of the agent’s execution capability before developing a willingness to rely on it. Users may integrate complex interface feedback features into a reliability-related evaluation. This interpretation aligns with the perspective of Glikson and Woolley (2020) that human trust in AI begins with a rational estimation of its competence. In the present study, the supported indirect pathways suggest an association among interaction-feature perceptions, perceived reliability, and human-agent trust. This finding is also consistent with prior evidence that excessive explainability may be linked to cognitive overload rather than increased trust when reliability support is insufficient (Poursabzi-Sangdeh et al., 2021). Therefore, if the agent’s interaction interface cannot help users translate technical features into reliability evaluations, technical advancement alone may struggle to produce sustained interaction trust. This interpretation suggests a possible implication for future work: explainability and intention-alignment cues may help users form more appropriate reliability judgments when interacting with generative AI agents.

The non-supported H7 pathway further suggests that perceived sense of agency may not be associated with trust through the same reliability-based pathway as perceived explainability and perceived intention alignment. A plausible explanation may lie in the psychological distinction between self-confidence and AI-confidence during task execution. Chong et al. (2022) showed that human confidence in AI and human confidence in themselves may evolve differently during AI-assisted decision-making. Building on this distinction, when semi-technical users are granted a high degree of agency, this experience may be more closely related to their self-confidence in managing the final task outcome than to their confidence in the AI’s intrinsic capabilities. In other words, semi-technical users may believe that the task can be completed successfully because they retain the ability to intervene and correct errors, not necessarily because the AI itself is perceived as more reliable. This interpretation is also consistent with Saffarizadeh et al. (2024), who showed that user-based steerability and autonomy may shape trust transference toward the AI system. When semi-technical users frequently exercise their agency to intervene or correct the AI’s intermediate reasoning, they may attribute the success of the final output partly to their own corrective actions rather than the AI’s competence. At the same time, the need for continuous intervention may also remind users that the system still requires oversight. Consequently, perceived sense of agency may function more as a safety mechanism for managing uncertainty than as a direct source of reliability-based trust. This interpretation may help account for why perceived reliability significantly mediated the associations involving perceived explainability and perceived intention alignment, but did not significantly mediate the relationship between perceived sense of agency and human-agent trust.

### 5.2 The moderating effects of perceived task complexity and domain self-efficacy

The positive moderation pattern involving perceived task complexity received support. The data suggest that when semi-technical users handle complex tasks characterized by high cognitive load and logical rigor, the positive association between perceived explainability and perceived reliability tends to be stronger, which is consistent with the research of Hermanns and Teubner (2025). A possible explanation is that in relatively simple development tasks, users may rely more on rapid result verification to form reliability evaluations (Buçinca et al., 2021). In contrast, under higher task complexity, the perceived cost of verifying the correctness of generated code or logic may also be higher (Vaithilingam et al., 2022), which may make explainability more salient for reliability judgments. In such contexts, perceived explainability may be viewed as less redundant and more relevant to reducing verification costs. Thus, the results suggest that perceived task complexity may be associated with a stronger relationship between explainability and perceived reliability.

Regarding domain self-efficacy, the empirical results showed a significant moderation pattern in the relationship between perceived intention alignment and perceived reliability, but in the opposite direction to the initial hypothesis. While H9 predicted a negative moderation pattern, the data showed a positive interaction: perceived intention alignment was more strongly associated with perceived reliability among users with higher domain self-efficacy. This unexpected positive pattern suggests that domain self-efficacy may be related to the evaluation process in generative AI-assisted development in a more complex way than initially assumed.

One tentative explanation is that high-efficacy users may possess more structured mental models of task goals and constraints prior to interaction (Schmutz et al., 2024). Because of these clearer internal standards, they may be more capable of evaluating whether the agent has correctly understood their goals, constraints, and task logic across multi-turn interaction. From this perspective, perceived intention alignment may become more salient for high-efficacy users. When a generative AI appears to grasp and follow these subtle constraints across multi-turn dialogues, high-efficacy users may interpret this alignment as evidence of the agent’s contextual understanding and collaborative fit, which may be associated with stronger perceived reliability. This interpretation is consistent with Inkpen et al. (2023), who showed that users’ baseline expertise, mental models of AI systems, and trust in recommendations jointly shape human-AI team performance. Their findings further suggest that highly proficient users are generally better able to discern when to follow AI recommendations. Conversely, if the AI deviates from their precise intent, high-efficacy users may be more capable of detecting such misalignment. Related evidence from Wang et al. (2023) shows that worker experience can shape how individuals benefit from and evaluate AI, with senior workers showing a relatively lower propensity to trust AI because of their broader task awareness. In contrast, low-efficacy users may lack explicit prior standards or may have difficulty formulating instructions with fine-grained constraints (Zamfirescu-Pereira et al., 2023). Under such conditions, low-efficacy users may focus more on surface-level output fluency and less on whether the agent has truly aligned with complex task intentions. As a result, their reliability judgments may be less sensitive to perceived intention alignment. This exploratory interpretation suggests that expertise does not necessarily reduce the importance of alignment cues; rather, in generative AI-assisted development, it may be associated with greater sensitivity to whether the agent can maintain cognitive and intentional fit during collaboration. However, this explanation should be treated cautiously and further validated in future research.

## 6 Limitations and future prospects

Adopting the perceptual perspective of semi-technical users, this study identified perceived explainability, perceived intention alignment, and perceived sense of agency as core antecedent variables. Using structural equation modeling, this study examined a theoretical model of trust formation for generative AI agents, with perceived reliability as a mediating variable and perceived task complexity and domain self-efficacy as moderating variables.

Nevertheless, several limitations should be acknowledged. First, the target population primarily focused on semi-technical users with recent experience in AI-assisted development, and other demographic parameters of the sample were not strictly controlled or stratified. Consequently, the generalizability of the findings may be subject to certain constraints. Future research should further segment and analyze specific sample characteristics. Second, the study used a cross-sectional self-report survey. Therefore, it captured users’ perceptual antecedents of trust formation rather than directly observing behavioral reliance or the mismatch between subjective trust and objective AI performance. In addition, the AI-assisted development context was based on participants’ recent experience rather than a standardized experimental task, and this experience may have varied across AI tools, task types, frequency of use, and skill levels. Therefore, perceived task complexity reflected subjective task evaluations rather than an objectively manipulated condition. Future research should use longitudinal or experimental designs with controlled task scenarios, objective performance indicators, and more clearly segmented user groups. Such designs could improve reproducibility, examine changes in trust and reliance over time, and determine whether these perceptual mechanisms differ across specific AI tools, task domains, and levels of user expertise. Third, although Harman’s single-factor test, one-factor CFA comparison, and a common latent method factor model were used as diagnostic and robustness checks, common method bias cannot be fully excluded because all focal variables were collected from the same questionnaire at a single point in time. Future research should consider multi-source, time-lagged, or behavioral data to further reduce and assess common method bias. In addition, although *N* = 312 was adequate for estimating the main SEM paths, the moderation analyses involving interaction effects increased analytical complexity and may require greater statistical power. Therefore, these interaction findings should be further validated with larger samples. Finally, the indirect association involving perceived sense of agency was not supported, and domain self-efficacy showed a positive moderation pattern opposite to the original hypothesis. Future research could examine whether perceived sense of agency is associated with trust through alternative psychological pathways and whether the positive moderating role of domain self-efficacy is replicated across different generative AI tasks and user groups.

## 7 Conclusion

By integrating perceived explainability, perceived intention alignment, and perceived sense of agency into the theoretical framework of human-AI trust, this study offers a cognitive perspective for understanding the trust formation process in generative AI-assisted development scenarios.

The study offers two main implications. First, it examines a cognitive model of trust formation in the context of generative AI, suggesting that perceived reliability may act as an important link between interaction features and trust attitudes when users interact with probabilistic AI agents. However, this mediation mechanism should be interpreted mainly in relation to perceived explainability and perceived intention alignment. The indirect association involving perceived sense of agency did not reach the conventional significance threshold, suggesting that user agency may be related to trust formation through other psychological routes rather than through perceived reliability alone. Second, this study expands the discussion of boundary conditions in trust formation. The findings suggest that perceived task complexity was associated with a stronger relationship between perceived explainability and perceived reliability. In addition, the results showed an unexpected positive moderating pattern of domain self-efficacy. Although the original hypothesis predicted a negative moderation pattern, higher domain self-efficacy was associated with a stronger positive relationship between perceived intention alignment and perceived reliability, suggesting the possibility that higher-efficacy users may be more sensitive to whether the agent accurately understands and follows their task intentions.

Based on these findings, this study offers the following practical implications. First, designers of generative AI-assisted development tools may consider improving how reasoning processes are represented in the interface. Clear explanations of problem-solving steps and logic may help users transform technical transparency into cognitive understandability, especially in complex tasks. Second, agent interaction design may prioritize precise alignment with user intent. For instance, providing anticipated code output or intermediate confirmation before full generation may help users evaluate whether the agent has correctly understood their goals and constraints. Finally, although perceived sense of agency was positively associated with perceived reliability, its reliability-based indirect association with trust was not statistically supported. Therefore, fine-grained control functions may be designed not simply as automatic trust-enhancing features, but as mechanisms that help users intervene, compare alternatives, correct outputs, and manage uncertainty during interaction.

## Data Availability

The datasets presented in this article are not readily available because the datasets generated and analyzed during the current study are not publicly available due to privacy and ethical restrictions but are available from the corresponding author on reasonable request. Requests to access the datasets should be directed to Yaoming Gong, 242592893@st.usst.edu.cn.
